# Emerging Therapeutics in COPD: Mapping Innovation to Treatable Traits

**DOI:** 10.1007/s00408-025-00844-0

**Published:** 2025-09-03

**Authors:** Mario Cazzola, Vanessa M. McDonald, Daiana Stolz, Paola Rogliani, Maria Gabriella Matera

**Affiliations:** 1 Unit of Respiratory Medicine, Department of Experimental Medicine, University of Rome ‘Tor Vergata’, Rome, Italy; 2 College of Health, Medicine and Wellbeing, University of Newcastle, Callaghan, NSW Australia; 3 Department of Respiratory and Sleep Medicine, John Hunter Hospital, New Lambton Heights, NSW Australia; 4 Clinic of Respiratory Medicine, Faculty of Medicine, University of Freiburg, Freiburg, Germany; 5 Unit of Pharmacology, Department of Experimental Medicine, University of Campania ‘Luigi Vanvitelli’, Naples, Italy

**Keywords:** Chronic obstructive pulmonary disease, Treatable traits, Emerging pharmacological therapies, Anti-inflammatory agents, Bronchodilators

## Abstract

Chronic Obstructive Pulmonary Disease (COPD) is a complex, heterogeneous condition characterized by diverse clinical phenotypes and underlying pathobiological mechanisms. Traditional “one-size-fits-all” management strategies have limited effectiveness in addressing this heterogeneity. The Treatable Traits (TTs) approach represents a precision medicine paradigm that targets specific, identifiable, and modifiable traits in individual patients, regardless of diagnostic labels. This paper explores the alignment between the TTs framework and emerging pharmacological therapies, with a particular focus on anti-inflammatory agents and bronchodilators currently under investigation. Each drug category is mapped to relevant TTs, such as eosinophilic or neutrophilic inflammation, corticosteroid resistance, chronic bronchitis, and frequent exacerbations. This review highlights the importance of biomarker-driven phenotyping and real-world data in designing TT-based clinical trials. It emphasizes challenges such as trait instability over time, comorbidity clustering, and trial design heterogeneity. Moreover, we advocate for incorporating digital health tools, long-term follow-up, and cost-effectiveness analyses to ensure translational relevance. In conclusion, integrating emerging therapies with the TTs approach holds substantial promise for personalizing COPD management, improving outcomes, and facilitating targeted drug development.

## Introduction

According to the 2025 Global Initiative for Chronic Obstructive Lung Disease (GOLD) strategy, chronic obstructive pulmonary disease (COPD) is a heterogeneous lung condition characterized by persistent respiratory symptoms and airflow limitation caused by abnormalities in the airways (e.g., bronchitis, bronchiolitis) and/or alveoli (e.g., emphysema) [1]. COPD results from an abnormal response to harmful substances, such as cigarette smoke, leading to structural damage, including emphysema, airway remodeling, and peribronchial fibrosis, and persistent inflammation involving macrophages, neutrophils, and lymphocytes. GOLD emphasizes that this inflammatory response is an amplification of the normal defense against chronic irritants, underscoring the multifactorial and complex nature of the disease [1]. This complexity is reflected in the broad spectrum of clinical, physiological, and biological features that vary significantly among individuals and evolve over time [2, 3]. The heterogeneity presents major challenges for diagnosis and management, limiting the effectiveness of conventional one-size-fits-all treatment approaches [4]. Current COPD therapies are largely supported by pivotal phase III randomized controlled trials (RCTs) that enroll large populations selected based on narrow criteria, typically the degree of airflow obstruction, smoking history, symptom burden, or exacerbation frequency [1]. While these studies have shaped evidence-based guidelines, they fall short of capturing the underlying complexity of COPD and offer limited support for personalized treatment approaches [5]. As a result, COPD management remains largely empirical despite growing interest in stratified care models. A promising alternative to traditional disease classification is the treatable traits (TT) paradigm, a label-free framework that aims to deconstruct chronic airway diseases into distinct, quantifiable, clinically meaningful, and modifiable components [6, 7]. Unlike conventional models based on static phenotypes or diagnostic categories [8], the TT approach targets specific, measurable traits as the focus of individualized interventions. This strategy acknowledges that patients often exhibit multiple overlapping traits, which may fluctuate in prominence over time due to disease progression, environmental exposures, comorbidities, or treatment effects. The TT model was initially developed to encompass a broad spectrum of chronic airway diseases, including asthma, COPD, and asthma-COPD overlap (ACO) [5]. However, the present manuscript focuses exclusively on physician-diagnosed COPD. All analyses, evidence, and therapeutic considerations pertain specifically to COPD, and references to asthma or ACO are made solely to contextualize the model’s origins and conceptual breadth [5, 6]. The flexibility of the TT model has led to its increasing recognition as a foundation for precision medicine in COPD, relevant both during stable disease and exacerbations [6]. It also supports the development of novel clinical trial designs, in which therapeutic interventions are evaluated based on trait-targeted efficacy rather than broad diagnostic categories. However, several practical challenges must be addressed before full-scale implementation is feasible.

The NOVELTY study, a large international, prospective, observational cohort, enrolled over 11,000 adults with physician-diagnosed asthma, COPD, or ACO from both primary and specialist care settings and followed them for three years [9]. It systematically collected data on clinical characteristics, disease progression, exacerbation history, healthcare utilization, and biomarker profiles. Among individuals classified as having COPD, participants exhibited an average of 5.4 coexisting traits, compared to 4.6 in those with asthma and 6.4 in the ACO group [10]. The most frequently observed trait combinations in COPD included non-reversible airflow limitation with emphysema, productive cough with environmental exposures, exacerbation proneness, and frequent reliever use. Importantly, early analyses revealed that a substantial proportion of patients with mild COPD (20.4%) reported at least one exacerbation in the preceding year, underscoring the limitations of conventional severity classifications in accurately predicting clinical risk [11]. In addition, blood eosinophil levels, often used as a surrogate for type 2 (T2) inflammation, did not consistently distinguish between asthma, COPD, and ACO groups, and showed poor correlation with clinical phenotype and exacerbation risk. In contrast, neutrophil counts increased with disease severity, raising questions about the utility of current inflammatory biomarkers in guiding individualized treatment decisions. These findings underscore the value of the TT approach while also illustrating its current limitations when applied to inflammatory traits.

It remains an open question how to determine which traits are most clinically significant, how they interact, and how they should be prioritized [12, 13]. While certain traits, such as airflow obstruction or exacerbation frequency, tend to remain stable across demographic and clinical subgroups, others, including symptom burden and biomarker levels, demonstrate substantial temporal variability and are sensitive to therapeutic interventions. This dynamic nature complicates their reliability as consistent endpoints in longitudinal research and precision medicine trials [12, 14]. Moreover, long-term observational data indicate that specific traits, such as chronic bronchitis, dyspnea, underweight, sarcopenia, and active smoking, are associated with accelerated lung function decline. Psychosocial traits, including depression and poor social support, have been linked to deteriorating health-related quality of life, particularly in older COPD populations [15].

Ultimately, while the TT paradigm offers a compelling framework to capture the clinical heterogeneity of COPD, its successful implementation demands rigorous evaluation of trait stability, biomarker validity, cross-disease generalizability, and the availability of effective, trait-targeted interventions [16].

## Evidence Supporting the Treatable Traits Approach in COPD

The TT approach shifts the focus of COPD management from a “one-size-fits-all” approach to individualized care, based on clinically meaningful, modifiable characteristics [6]. This strategy acknowledges the heterogeneity of COPD and emphasizes treating the whole patient rather than solely focusing on airflow limitation.

An integrated analysis of a non-randomized COPD study and a RCT in severe asthma identified recurrent chest infections, dysfunctional breathing, poor inhaler technique, systemic inflammation, and depression as key TTs negatively affecting health-related quality of life (HRQoL) [17]. Treating these traits with targeted therapies, such as statins for systemic inflammation and corticosteroids for eosinophilic inflammation, led to substantial improvements in HRQoL compared to standard care. In contrast, smaller gains were observed when treating anxiety, obesity, and exercise intolerance.

A systematic review [18] of six studies involving 1109 patients with COPD examined 13 to 36 traits per study [19–24] and determined that TT interventions significantly improved several clinical outcomes. These benefits included improvements in St. George’s Respiratory Questionnaire scores, as well as improvements in dyspnea, anxiety, and depression. There was also a reduction in hospital admissions due to exacerbations and a modest improvement in functional capacity as measured by the six-minute walk test.

The presence of extrapulmonary traits, including fatigue, psychological distress, and reduced physical capacity, was found to enhance responsiveness to pulmonary rehabilitation (PR). In a study where patients presented with an average of three TTs, improvements were seen in five of nine outcome domains post-PR [25]. The presence of these traits enhanced the probability of a positive PR response by 4 to 20 times.

The 2025 GOLD report acknowledges the relevance of TTs, particularly dyspnea and exacerbations, in its treatment algorithm [1]. The report also recognizes the importance of addressing behavioral, extrapulmonary, and social factors when present.

Two recent publications [26, 27] provided more information on implementing the TT model in COPD care (Table 1). Thomas and Beasley [26] proposed a framework that categorizes traits into pulmonary, extrapulmonary, and behavioral domains, providing practical management strategies. Agustí et al. [27] emphasized the feasibility of the model in primary care settings and identified seven key traits central to COPD management: airflow limitation, eosinophilic inflammation, poor adherence, incorrect inhaler technique, smoking, low body mass index (BMI)/obesity, and anxiety/depression.

**Table 1:** A comprehensive framework for managing COPD by identifying and targeting specific treatable traits

Treatable trait	Thomas & beasley [26]	Agusti et al. [27]
Airflow obstruction	Maintenance: LABA/LAMA Rescue: SABA/SAMA/rapid-acting LABA	LABAs, LAMAs
Eosinophilic inflammation	Corticosteroids, anti-IL-5, -13, -4 monoclonal antibody therapy	ICS-LABA
Adherence issues	Education, treatment simplification, regular follow-up	Education, treatment simplification, regular follow-up
Inhaler technique	Education including demonstration and regular reassessment	Education and correction of inhaler technique
Smoking	Smoking cessation counseling ± pharmacotherapy	Smoking cessation support
Low BMI/Obesity	Overweight/obesity: Caloric restriction, exercise, bariatric surgery and pharmacotherapy	Nutritional counseling and interventions (diet, exercise, extra meal a day when BMI is low)
Anxiety and depression	Pharmacotherapy (i.e., anxiolytics/antidepressants), breathing retraining, cognitive behavioral therapy	Screening and treatment with pharmacological and psychological interventions
Neutrophilic inflammation	Macrolides, tetracyclines, and roflumilast	
Chronic Bacterial infection	Antibiotics and tailored antibiotic written action plan for infections	
Systemic inflammation	Statins	

BMI, body mass index; ICS, inhaled corticosteroid; IL, interleukin; LABA, long-acting beta agonist; LAMA, and long-acting muscarinic antagonist; SABA, short-acting beta agonist; SAMA, short-acting muscarinic antagonist

Both papers support using bronchodilators to manage airflow limitation and inhaled corticosteroids (ICSs) to treat eosinophilic inflammation (sputum eosinophils ≥ 3% and/or fractional exhaled nitric oxide [FeNO] ≥ 30 ppb and/or blood eosinophils ≥ 0.3 × 10^9^ cells L^−1^) [26, 27]. This emphasizes their central role in trait-targeted pharmacotherapy. Furthermore, adherence to treatment and correct inhaler technique were identified as essential, modifiable factors that influence therapeutic success. Anxiety and depression, recognized as significant determinants of poor clinical outcomes, warrant a dual approach involving both pharmacological and psychological interventions. It is noteworthy that Agustí et al. have emphasized the significance of nutritional support for addressing low BMI [27], a factor not included in the framework proposed by Thomas and Beasley [26]. Conversely, Thomas and Beasley [26] have considered traits such as neutrophilic inflammation (sputum neutrophils ≥ 61%), chronic bacterial infection, and systemic inflammation (leukocyte count > 9 × 10^9^ cells L^−1^ or high-sensitivity C-reactive protein [CRP] > 3 mg L^−1^), underscoring the need for long-term antibiotics or broader management approaches. Furthermore, Agustí et al. have emphasized the feasibility of implementing the TT approach in primary care [27], while Thomas and Beasley have endorsed its applicability in both primary and secondary care settings [26].

## The Need for Novel Therapies in COPD

The prevailing approach to the treatment of COPD continues to rely heavily on the utilization of bronchodilators and corticosteroids [1]. Consequently, it is not surprising that the treatment of TT is also based mainly on the utilization of these two classes of drugs [23, 24]. However, current pharmacological strategies remain inadequate for a significant subset of patients.

Traditionally, COPD is characterized by chronic neutrophilic inflammation. However, a growing body of evidence has revealed a wider spectrum of inflammatory profiles. Approximately 20%–40% of patients demonstrate eosinophilic airway inflammation, and some exhibit a mixed eosinophilic-neutrophilic pattern [28]. Additionally, macrophages, CD4^+^ and CD8^+^ T cells, and B lymphocytes contribute variably to disease pathogenesis [28]. This suggests that, even within a single inflammatory phenotype, the dominance of specific mediators may vary between individuals. This complex immunopathology implies that a uniform anti-inflammatory strategy is unlikely to be effective for the entire COPD population [29].

Although ICSs remain a cornerstone of anti-inflammatory therapy, they exhibit limited efficacy in most COPD patients, particularly those with predominant neutrophilic inflammation or ongoing tobacco exposure [30, 31]. Similarly, although bronchodilators are effective in controlling symptoms, they do not consistently address the various pathobiological mechanisms that contribute to airflow limitation [32]. These limitations highlight the urgent need for novel pharmacological agents that can precisely target disease traits, offering a personalized, potentially more effective approach to disease modification.

## Aligning Drug Development for COPD with the Treatable Traits Approach

Over the past two decades, drug development for COPD has increasingly focused on disrupting the recruitment and activation of immune cells, as well as neutralizing the inflammatory mediators they release [29, 33, 34]. Structural and inflammatory cells within the lung produce a complex network of chemokines, cytokines, lipid mediators, and growth factors that sustain chronic inflammation and tissue remodeling. Modulating these pathways therapeutically offers a rational strategy for addressing specific inflammatory traits.

In this context, the TT framework is a vital link between drug discovery and clinical application. By associating pharmacological candidates with specific traits, such as eosinophilic inflammation, neutrophilic dominance, systemic inflammation, or corticosteroid resistance, researchers and clinicians can more effectively stratify patient populations and tailor interventions accordingly.

## Emerging Anti-inflammatory Agents

Several classes of emerging anti-inflammatory agents demonstrate the integration of TTs into COPD drug development, enabling more targeted and personalized therapy (Table 2).

**Table 2:** - Overview of the current scenery of emerging anti-inflammatory therapies in COPD, highlighting their targeted treatable traits and development status

Drug class	Representative agents	Linked Treatable traits	Clinical development status
Inhibitors of the recruitment and activation of cellular inflammatory components and antagonists of the products of these components			
PDE4 inhibitors	Tanimilast	Neutrophilic inflammation, chronic bronchitis, frequent exacerbations	Phase III
Dual PDE3/4 inhibitors	Ensifentrine	Airway obstruction, neutrophilic airway inflammation, exacerbation-prone phenotype, chronic bronchitis phenotype	FDA Approved
CXCR2 antagonists	Ladarixin	Neutrophilic airway inflammation, frequent exacerbations	Phase II
p38 MAPK inhibitors	Acumapimod, CHF6297, PUR 1800	Corticosteroid resistance, systemic inflammation, frequent exacerbations	Phase II
PI3K-δ inhibitors	CHF6523	Corticosteroid resistance, neutrophilic inflammation, exacerbation-prone phenotype	Clinical development halted due to lack of
Selectin antagonists	Bimosiamose	Neutrophilic airway inflammation, vascular/systemic inflammation	Early-phase trials in asthma; limited data in COPD
IL-17 inhibitors	CNTO 6785	Neutrophilic inflammation, Th17-mediated immune response	Phase II; limited efficacy observed
Anti-IL-5 therapies	Mepolizumab, Benralizumab	Eosinophilic inflammation, frequent eosinophilic exacerbations	Approved for eosinophilic COPD
Anti-IL-4/IL-13 therapies	Dupilumab	T2 inflammation, eosinophilic phenotype, asthma-COPD overlap	FDA Approved
Anti-TSLP therapies	Tezepelumab	T2 inflammation, eosinophilic-prone phenotype, corticosteroid unresponsive patients	Phase III; did not meet primary endpoint
IL-33 inhibitors	Astegolimab, Itepekimab, Tozorakimab	T2 inflammation, allergic inflammation, eosinophilic COPD	Mixed results; further evaluation needed
Therapies that antagonize the products of the cellular components of inflammation in COPD			
MMP Inhibitors	Various agents	Emphysema progression, airway remodeling	Phase III trials failed; development discontinued
NE Inhibitors	AZD9668, MR889	Neutrophilic inflammation, mucus hypersecretion, chronic bronchitis	Clinical trials showed limited efficacy
DPP1 Inhibitors	Brensocatib	Neutrophilic inflammation, exacerbation-prone phenotype	Effective in bronchiectasis; not yet studied in COPD
Recombinant Human AAT	INBRX-101	AATD	Phase I
Gene Therapy for AATD	BEAM-302	AATD, emphysema progression	Phase I/II

AAT, alpha-1 antitrypsin; AAT deficiency, AATD; CXCR2, C-X-C Motif Chemokine Receptor 2; DPP1, dipeptidyl peptidase 1; IL, interleukin; MAPK, mitogen-activated protein kinase; MMP, matrix metalloproteinase; NE, neutrophil elastase; PDE, phosphodiesterase; PI3K-δ, phosphoinositide 3-kinase-δ; Th, T helper; TSLP, thymic stromal lymphopoietin; T2, type 2

For example, phosphodiesterase (PDE) 4 inhibitors, such as tanimilast, reduce neutrophilic inflammation and are ideal for patients with the chronic bronchitis phenotype and a high risk of exacerbations [35]. Dual PDE3/4 inhibitors, such as ensifentrine, offer combined anti-inflammatory and bronchodilator properties. They address multiple TTs, including neutrophilic airway inflammation and the exacerbation-prone and chronic bronchitis phenotypes [36]. Other promising agents are being aligned with specific pathobiological traits. C-X-C motif chemokine receptor 2 (CXCR2) antagonists aim to reduce neutrophil recruitment and activation. This makes them relevant for patients with neutrophilic airway inflammation and exacerbation-prone phenotypes [37]. p38 mitogen-activated protein kinase (MAPK) inhibitors are intended for patients with systemic inflammation or corticosteroid resistance. These traits often coincide with high CRP levels and a poor response to standard therapy [38]. Similarly, phosphoinositide 3-kinase (PI3K)-δ inhibitors are being developed for individuals with corticosteroid insensitivity driven by oxidative stress and neutrophilic inflammation, especially in individuals with comorbid atherosclerosis [39, 40].

Biologic therapies represent a significant advancement in TT-based interventions. The most clearly defined TT-targeted biologics are anti-interleukin (IL)-5 agents, such as mepolizumab. These agents reduce eosinophilic inflammation and exacerbations in patients with blood eosinophil counts of at least 300 cells/μL [41]. This threshold defines the trait and identifies frequent eosinophilic exacerbations and eosinophilic inflammation as the primary TTs for these agents [27, 42, 43]. Other biologics target broader or overlapping T2 inflammatory pathways. Anti-IL-4/IL-13 therapies, such as dupilumab, are effective in patients with T2-high COPD. This condition is characterized by elevated FeNO, eosinophilia, and ACO. These therapies target TTs, such as mucus hypersecretion, airway remodeling, and comorbid asthma [44]. Anti-thymic stromal lymphopoietin (TSLP) agents (e.g., tezepelumab) aim to dampen multiple inflammatory cascades and may benefit steroid-unresponsive patients or those with eosinophilic traits. However, clinical efficacy remains inconsistent [45, 46]. Biologics targeting IL-33 (e.g., astegolimab, itepekimab, tozorakimab) may be useful for patients with allergic comorbidities or overlapping asthma traits being focused on T2 inflammation, addressing TTs such as eosinophilic COPD, ACO, and exacerbation risk [47–49]. However, current data show variable impact on exacerbation rates, highlighting the need for refined patient selection. Anti-IL-17 therapies, which modulate Th17-driven immune responses, are being explored as potential treatments for COPD patients with neutrophilic inflammation, particularly corticosteroid-insensitive patients [41]. Although clinical application is still in the early stages and patient selection remains challenging, this class may align with TTs such as neutrophilic airway inflammation and phenotypes that are prone to exacerbation and unresponsive to standard anti-inflammatory therapies [41].

Protease activity plays a key role in the pathogenesis of COPD, particularly in emphysema and chronic bronchitis [29]. This makes it a relevant focus within the TTs framework. Several emerging and investigational therapies target this mechanism. Matrix metalloproteinase (MMP) inhibitors aim to limit extracellular matrix degradation and airway remodeling, which aligns with the TT of emphysema progression, especially in patients with imaging-confirmed disease. Despite promising preclinical results [29], no MMP inhibitor has succeeded in Phase III trials due to efficacy and safety limitations [50, 51]. Neutrophil elastase (NE) inhibitors target neutrophilic inflammation, mucus hypersecretion, and chronic bronchitis [52, 53]. These traits are frequently observed in patients with a high neutrophil burden and increased sputum production. Although clinical efficacy has been limited thus far, partly due to delivery issues and patient heterogeneity, NE inhibition remains a biologically plausible and worthwhile strategy for further investigation [29, 54]. Dipeptidyl peptidase-1 (DPP1) inhibitors, such as brensocatib, suppress the activation of neutrophil serine proteases and may reduce neutrophil-mediated tissue damage [55]. While these agents have not yet been studied in COPD, they have shown promise in bronchiectasis and may be valuable for treating phenotypes associated with neutrophilic inflammation and exacerbation, particularly in patients with bronchiectasis overlap [56]. Lastly, α1-antitrypsin (AAT) replacement therapy is an excellent example of a precision medicine approach. AAT deficiency (AATD) is a clearly defined and measurable TT, and its treatment with intravenous augmentation therapy aims to counteract unopposed proteolytic activity. This treatment reduces the rate of emphysema progression and lung function decline in eligible individuals [57–59]. Alternative administration routes are under investigation. Although inhaled AAT is theoretically appealing due to its direct delivery to the lungs, it has demonstrated limited efficacy in clinical trials [57]. However, preclinical data indicate that pulmonary administration and PEGylation (to prolong half-life) could increase lung exposure and anti-inflammatory effects [60]. Additionally, recombinant and plant-derived AAT products are in the early stages of clinical development to address the cost and supply limitations of plasma-derived products [61]. INBRX-101, a recombinant human AAT-Fc fusion protein, is under development [62]. It is a promising alternative to plasma-derived AAT, with the potential for improved pharmacokinetics and supply. Gene therapy for AATD-associated COPD is under active investigation. Emerging therapies such as clustered regularly interspersed short palindromic repeats (CRISPR) base-editing candidates like BEAM-302 represent a next-generation approach that aims to directly correct the pathogenic PiZ mutation in the SERPINA1 gene at the DNA level. BEAM-302 is being evaluated in an ongoing first-in-human clinical trial (NCT06389877) for its ability to increase circulating functional AAT levels or reduce mutant Z-AAT protein in humans with lung involvement.

## Novel Bronchodilators

A growing body of research highlights the potential of novel bronchodilator classes that alleviate airflow limitation and modulate inflammatory pathways. These advancements align with the TTs framework, which emphasizes phenotype-driven, targeted interventions for COPD (Table 3). Of the several emerging bronchodilator classes [32, 63, 64], three stand out for their mechanistic relevance to TTs.

**Table 3:** – Overview of the current scenery of emerging bronchodilators in COPD, highlighting their targeted treatable traits and development status

Drug class	Representative agents	Linked treatable traits	Clinical development status
TAS2R agonists	TAS2R14 agonist-28.1, flufenamic acid	Airflow limitation, bronchial hyperresponsiveness	In preclinical phase; activation of TAS2R on airway smooth muscle induces bronchodilation via a unique mechanism. Clinical development awaited
EP4R agonists	PGE₂ analogs targeting EP4R	Bronchoconstriction, airway inflammation, airway remodeling	EP4R activation relaxes human airway smooth muscle and limits fibrosis/inflammation. Early preclinical; potential dual bronchodilation + anti-inflammatory action
ROCK inhibitors	Fasudil, ripasudil, netarsudil	Bronchial hyperresponsiveness, pulmonary hypertension	ROCK inhibition reduces smooth muscle contraction and vascular remodeling. Approved for pulmonary hypertension in Asia; COPD applications are preclinical/early phase
Calcilytics	Ca^2^⁺-sensing receptor antagonists	Bronchial hyperresponsiveness, potential neuro-immune modulation	Preclinical data suggest reduced airway responsiveness via Ca^2^⁺ receptor blockade. Clinical development status: none available for COPD, still speculative
PPAR-γ agonists	Rosiglitazone, pioglitazone	Neutrophilic inflammation, systemic inflammation, airway remodeling, mucus	In vitro and animal models show reduced TNF-α/IL-8, enhanced neutrophil clearance, antifibrotic effects. Clinical trials in COPD pending
Relaxin Receptor 1 agonists	LY3540378, serelaxin	Fibrosis, pulmonary vascular remodeling	Antifibrotic and vasodilating effects reported in other models, but no clinical COPD data currently
Soluble Guanylyl Cyclase activators	Riociguat, runcaciguat	Pulmonary hypertension, vascular inflammation/oxygenation impairment	Riociguat approved for pulmonary hypertension; agents improve vascular tone and pulmonary hemodynamics. COPD use still experimental
Pepducins (biased GPCR modulators)	β₂-adrenoceptor ICL₃ pepducins	Airflow limitation (bronchodilation), reduced GPCR desensitization	Preclinical studies show Gs-biased signaling to promote bronchodilation and reduced tachyphylaxis. Human trials not yet initiated

EP4R, E-prostanoid receptor 4; GPCR, G protein-coupled receptor; ICL, intracellular loop; IL, interleukin; PGE₂, prostaglandin E₂; PPAR-γ, peroxisome proliferator-activated receptor-γ ROCK, Rho kinase; TAS2R, bitter taste 2 receptor; TNF-α, tumor necrosis factor-α

Bitter-taste receptor (TAS2Rs) agonists activate TAS2Rs in airway smooth muscle, inducing bronchodilation via calcium-mediated signaling independent of β_2_-adrenoceptors [65, 66]. Preclinical evidence suggests that TAS2R agonists have additional anti-inflammatory properties [67], making them candidates for treating conditions such as airflow limitation and bronchial hyperresponsiveness. However, clinical translation remains limited due to challenges in receptor selectivity and formulation.

Rho kinase (ROCK) inhibitors reduce smooth muscle tone by inhibiting calcium sensitization, and exhibit vasodilatory and anti-inflammatory effects [32, 63, 68]. These properties align with TTs such as airway hyperresponsiveness, pulmonary hypertension, and vascular remodeling, particularly in advanced disease.

Pepducins are intracellular modulators of G protein-coupled receptor signaling that may enhance β_2_-adrenoceptor-mediated bronchodilation while minimizing desensitization [69–71]. This pharmacological profile suggests their usefulness in treating two critical TTs, bronchodilator tolerance and persistent airflow obstruction that does not respond to standard therapy.

## New Drugs that Protect Against Lung Damage and Help Regenerate Tissue

Lung damage in the context of harmful inhalation exposure or COPD can be conceptualized as a TT by targeting the underlying mechanisms of injury, inflammation, and impaired regeneration, and by leveraging emerging pharmacologic and regenerative therapies.

Experimental pharmacologic strategies to reduce lung damage after harmful inhalation focus on limiting oxidative stress, inflammation, and early fibrosis (Table 4). In preclinical models, edaravone combined with dexamethasone has shown synergistic effects in attenuating smoke-induced lung injury by reducing reactive oxygen species, inflammatory cytokines, and apoptosis, while improving antioxidant enzyme activity and histopathology in rats [72]. Tranilast, an anti-inflammatory and antifibrotic agent, has demonstrated protective effects against acute respiratory distress syndrome and early pulmonary fibrosis following smoke inhalation in animal models, reducing histopathological lung injury, decreasing pro-inflammatory cytokines (IL-1β, TNF-α, TGF-β1), alleviating oxidative stress, and promoting proliferation of alveolar epithelial and endothelial cells [73]. Additionally, inhaled antioxidant carriers, such as ligustrazine-loaded covalent cyclodextrin frameworks, have demonstrated efficacy in mitigating acute lung injury in rat models [74]. These carriers significantly reduce inflammation, oxidative stress, and lung damage. Mechanistic evidence suggests modulation of the nuclear factor erythroid 2-related factor 2/nuclear factor kappa B pathway.

**Table 4:** New drug approaches for mitigating lung injury and promoting tissue repair

Category	Drug/Therapy	Mechanism/Effect	Model/Notes
Pharmacologic strategies to reduce lung damage	Edaravone + Dexamethasone	Synergistic effect reducing reactive oxygen species, inflammatory cytokines, apoptosis; improves antioxidant enzymes and histopathology	Preclinical rat model of smoke-induced lung injury
	Tranilast	Anti-inflammatory and antifibrotic; reduces lung injury, pro-inflammatory cytokines (IL-1β, TNF-α, TGF-β1), oxidative stress; promotes alveolar cell proliferation	Animal models of smoke inhalation-induced ARDS and early pulmonary fibrosis
	Ligustrazine-loaded covalent cyclodextrin carriers	Inhaled antioxidant delivery; reduces inflammation, oxidative stress, lung damage; modulates Nrf2/NF-κB pathway	Rat models of acute lung injury
Regenerative therapies	P63 + lung progenitor cell transplantation	Stem cell-based therapy; improves gas transfer capacity and exercise tolerance; favorable safety	Early clinical trials in COPD patients
	DPP4 inhibitors (e.g., NZ-97)	Expands type 2 alveolar epithelial cells; promotes regenerative repair	Mouse models of lung injury
	Prostanoid receptor ligands (EP and IP agonists)	Restores alveolar epithelial progenitor function; promotes lung regeneration	Cigarette smoke-induced COPD mouse models
	Am80-encapsulated nanoparticles (synthetic retinoic acid)	Repairs alveolar destruction; improves lung function	Mouse model of elastase-induced emphysema

DPP4, dipeptidyl peptidase 4; IL, interleukin; NF-κB, nuclear factor kappa B; Nrf2, nuclear factor erythroid 2-related factor 2; TGF-β1, transforming growth factor-beta1; TNF-α, tumor necrosis factor-α

For the regeneration of lung tissue in COPD, several experimental approaches are under investigation. Stem cell-based therapies, including autologous transplantation of P63 + lung progenitor cells, have shown early clinical promise, with improvements in gas transfer capacity and exercise tolerance in COPD patients, and a favorable safety profile [75]. Pharmacologic agents targeting alveolar repair include DPP4 inhibitors (e.g., NZ-97), which selectively expand type 2 alveolar epithelial cells and promote regenerative repair in mouse models of lung injury [76]. Prostanoid receptor ligands (EP and IP receptor agonists) have demonstrated the ability to restore alveolar epithelial progenitor function and promote lung regeneration in cigarette smoke-induced COPD models [77]. Am80-encapsulated nanoparticles (a synthetic retinoic acid) have also shown efficacy in repairing alveolar destruction and improving lung function in a mouse model of elastase-induced emphysema [78].

While these approaches are promising, none are yet established in clinical guidelines, and further large-scale trials are needed to confirm efficacy and safety in humans.

## Designing a Treatable Traits-Focused RCT in COPD

Conducting a TT-focused RCT in COPD poses unique methodological and logistical challenges due to the disease’s heterogeneity and the evolving precision medicine paradigm [79]. Unlike traditional RCTs, TT trials require stratification based on discrete, modifiable traits that, as previously stated, often coexist in the same patient, with an average of five TTs per individual [10]. The absence of a clear hierarchy among traits, their dynamic expression, and variation across care settings further complicate trial design [13, 14].

There are still limited prospective TT-focused studies, and much of the current evidence is derived from post hoc analyses. It is critical to define stable traits, and a run-in period of 3–6 months is recommended to confirm TT persistence before randomization. TT clusters, identified via machine learning and clinical consensus, may offer a pragmatic solution by allowing stratification and randomization by cluster rather than by individual traits.

Adaptive platform trials (e.g., REMAP-CAP [80] and I-SPY 2 [81]) provide a flexible framework that allows for the parallel evaluation of multiple TTs under a unified protocol. Treatment arms can be modified as new evidence emerges. An ongoing adaptive trial in sepsis (NCT06381661) exemplifies this approach and may serve as a model for future COPD research.

Endpoints should explicitly link to targeted traits by incorporating TT-specific outcomes (e.g., sputum volume and eosinophil counts) and composite measures reflecting broader clinical benefits. The use of biomarker-based and digital tools, such as FeNO testing, home spirometry, and symptom-tracking apps, enhances real-time monitoring and treatment precision [79].

Engaging with regulators is crucial for validating novel endpoints and accepting composite measures [79]. To ensure generalizability, recruitment should aim for diverse populations across healthcare systems. Economic evaluations and quality-of-life assessments should also be included, particularly for resource-intensive interventions (e.g., biologics and PR) [26, 27]. A minimum follow-up period of one to three years is recommended to evaluate the durability of the effects and the evolution of the traits.

**Fig. 1:**
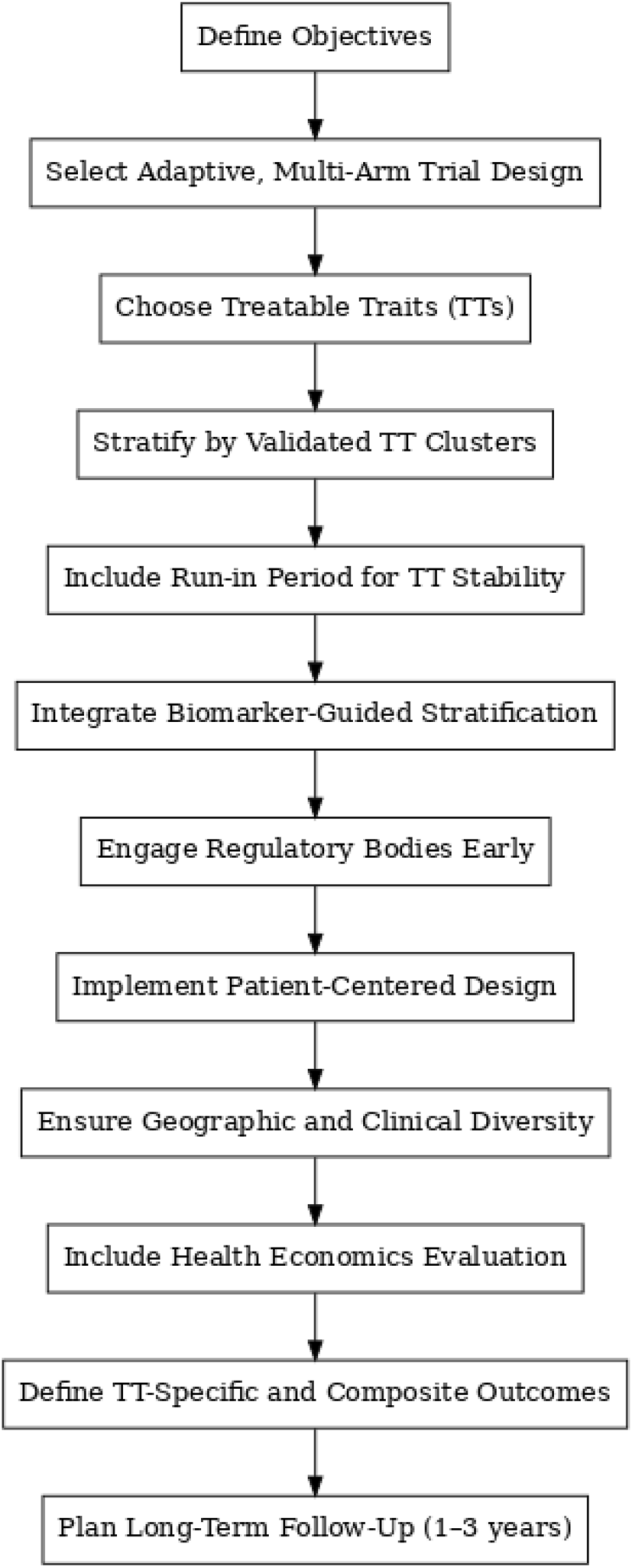
TT-focused RCT design in COPD. Flowchart of key design elements

When designed with these elements in mind (Table 5, Fig. 1), RCTs focused on TTs can offer high internal validity, external relevance, and actionable insights for personalized COPD care.

**Table 5:** Key design elements for a TT-focused RCT in COPD

Component	Suggested approach	Rationale
Trial design	Adaptive, multi-arm platform trial	Accommodates multiple TTs and interventions; allows dynamic modification based on interim data
Patient stratification	Stratify by validated TT clusters (e.g., based on NOVELTY data)	Addresses the co-occurrence of TTs; improves homogeneity within groups
TT selection	Prioritize prevalent and impactful TTs (e.g., exacerbation risk, chronic cough, airflow limitation)	Ensures clinical relevance and feasibility
TT stability check	Include a run-in period (e.g., 3–6 months) to confirm trait persistence	Reduces misclassification; ensures more stable endpoints
Outcome measures	Use TT-specific endpoints and composite outcomes (e.g., TT resolution, symptom scores, biomarkers)	Captures both targeted and holistic treatment effects
Biomarker integration	Employ biomarker-guided stratification and monitoring (e.g., eosinophils, FeNO, imaging, digital tools)	Enhances precision and objectivity in measuring TT activity and response
Regulatory alignment	Engage regulators early to agree on endpoints, biomarker validation, and combination outcomes	Increases likelihood of trial approval and translation
Patient-centered design	Include patient-reported outcomes and involve patient advisory boards	Improves relevance and acceptability of interventions
Generalizability	Recruit from diverse geographic and care settings	Enhances external validity and applicability
Health Economics component	Integrate cost-effectiveness and QALY analyses	Supports decision-making and reimbursement justification
Follow-Up duration	Include long-term follow-up (1–3 years post-treatment)	Assesses durability of effects and TT evolution

FeNO, fractional exhaled nitric oxide; QALY, quality-adjusted life year

## Conclusion

The TT framework is a compelling strategy for addressing the clinical and biological heterogeneity of COPD. Rather than redefining the disease, the TT framework reframes how we approach its complexity by prioritizing precision over categorization and personalizing care based on modifiable, measurable traits [42]. This paradigm is supported by an increasing number of emerging pharmacological agents, ranging from new anti-inflammatory agents [29, 33, 34] to novel bronchodilators [32, 63. 64]. Each agent is aligned with a specific trait, such as eosinophilic inflammation, airway limitation, emphysema progression, or bronchodilator tolerance.

However, the promise of TT-based approach to COPD treatment will remain largely theoretical without methodological innovation in trial design [42, 79]. Future clinical research must evolve to embrace this complexity by integrating biomarker-driven stratification, trait-cluster selection, and adaptive platform methodologies that reflect the dynamic nature of COPD. These designs must test therapeutic efficacy and validate TTs as actionable clinical tools.

Ultimately, integrating the TT approach into drug development and routine practice is a critical step toward a more personalized, biologically based model of care. Aligning treatment with underlying mechanisms rather than broad diagnostic categories has the potential to improve outcomes for COPD patients.

## Data Availability

No datasets were generated or analyzed during the current study.
